# Cosegregation analysis following an excellent response to olaparib in a pancreatic cancer patient carrier of *BRCA2*:c.7892 T > C variant enables its reclassification from VUS to pathogenic

**DOI:** 10.1038/s44276-026-00206-0

**Published:** 2026-02-16

**Authors:** Ksenija Strojnik, Ana Blatnik, Mateja Krajc, Aleksander Novaković, Marija Ignjatović, Janja Ocvirk, Vida Stegel, Petra Škerl, Gašper Klančar, Srdjan Novaković, Vita Šetrajčič Dragoš

**Affiliations:** 1 Department of Clinical Cancer Genetics, Institute of Oncology Ljubljana, Ljubljana, Slovenia; 2 University of Ljubljana, Ljubljana, Slovenia; 3 Division of Medical Oncology, Institute of Oncology Ljubljana, Ljubljana, Slovenia; 4 Department of Molecular Diagnostics, Institute of Oncology Ljubljana, Ljubljana, Slovenia

## Abstract

Identification of variants of uncertain significance (VUS) presents a great challenge in oncogenetics, especially in the era of personalised cancer treatment. We present a metastatic pancreatic cancer patient, referred for predictive genetic testing for treatment with poly(ADP-ribose) polymerase (PARP) inhibitors, in whom a rare missense BRCA2:c.7892 T > C p.(Leu2631Pro), located in the DNA-binding domain, was identified. Extensive family history of cancers as well as data on other carriers, identified in our laboratory database of tested individuals, suggested hereditary breast and ovarian cancer (HBOC) syndrome. However, the variant could only be formally classified as a VUS at the time. In this exceptional case, an ad hoc board of experts was formed and proposed the patient be offered PARP inhibitors before time-consuming cosegregation analysis and formal reclassification of the VUS to pathogenic/likely pathogenic (P/LP) were completed. After a partial response to platinum-based chemotherapy, the patient consented to maintenance with olaparib and a 48-months long complete response was observed. Herein, we also present a formal reclassification of the variant BRCA2:c.7892 T > C from VUS to pathogenic after the completion of extensive cosegregation analysis in 71 members of a single large family originating from a specific northeastern region of Slovenia.

## Introduction

Identification of variants of uncertain significance (VUS) presents a great challenge in oncogenetics, especially when there is a high likelihood of pathogenicity, as VUS should not be used in clinical decision making [1]. Multiple lines of evidence, such as additional functional studies or cosegregation analysis, may be required for accurate reclassification of VUS, most often to benign/likely benign (B/LB), or more seldomly to pathogenic/likely pathogenic (P/LP) [2–5]. This process can be very time-consuming. In the past, genetic testing for germline P/LP variants in cancer predisposition genes was mostly performed for personalised surveillance and risk-reducing strategies, and in such cases there was usually ample time for VUS reassessment. In the era of personalised cancer treatment, germline P/LP variants in *BRCA1* and *BRCA2* genes are important as predictive biomarkers for platinum-based chemotherapy and poly(ADP-ribose) polymerase (PARP) inhibitors [6]. Rapid and accurate genetic analysis and variant interpretation are of utmost importance in such cases. A protracted process of variant reclassification can lead to cancer patients missing potential benefits of targeted therapy.

Herein, we present a metastatic pancreatic cancer patient referred for predictive genetic testing for treatment with PARP inhibitors, in whom a rare missense VUS *BRCA2*:c.7892 T > C p.(Leu2631Pro), located in the DNA-binding domain, was identified. Extensive personal and/or family history of breast, pancreatic, ovarian and prostate cancers in other carriers of the VUS, identified through our laboratory database of tested individuals, suggested hereditary breast and ovarian cancer (HBOC) syndrome. Due to a high likelihood of variant pathogenicity, an ad hoc board of experts was formed and proposed the patient be offered PARP inhibitors before the time-consuming cosegregation analysis and reclassification of the VUS to P/LP was completed. Also, a formal reclassification process of the variant *BRCA2*:c.7892 T > C from VUS to P after extensive cosegregation analysis a single large Slovenian family is reported.

## Methods

### Genetic testing

**Fig. 1:**
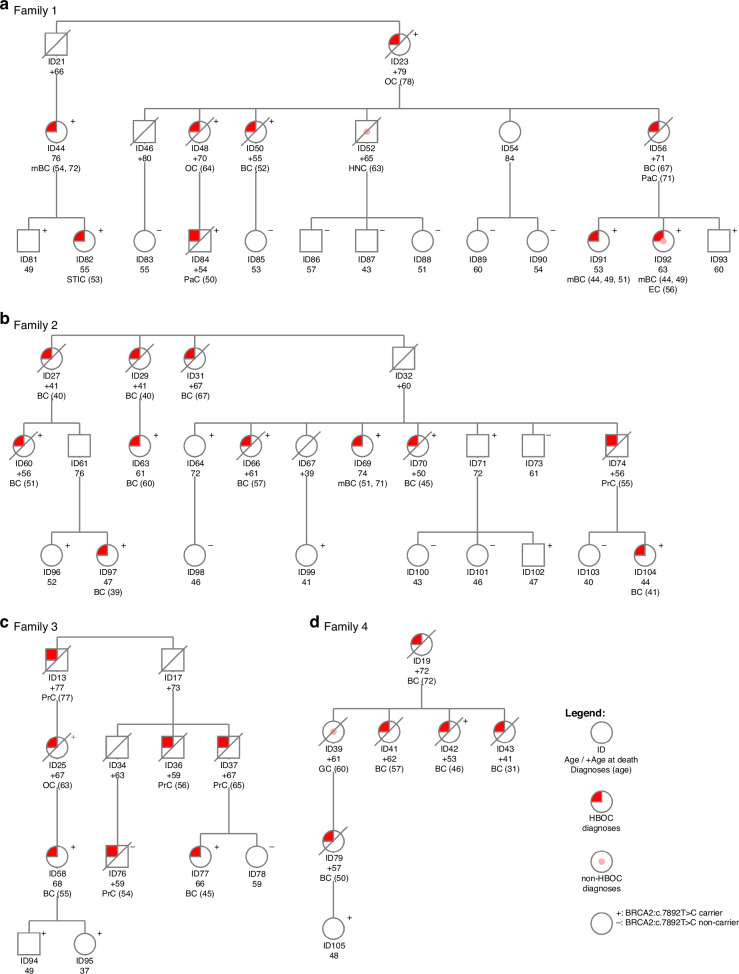
Pseudonymised pedigrees of the four initially identified families of carriers of BRCA2:c.7892 T > C germline variant. **a** Pedigree of the family showing the proband, in whom the BRCA2 variant was first identified. **b**, **c**, **d** Pedigrees of additional families with BRCA2:c.7892 T > C. Squares represent males and circles females. HBOC cancer diagnoses: BC breast cancer, mBC multiple breast cancers, OC ovarian cancer, PaC pancreatic cancer, PrC prostate cancer, STIC serous tubal intraepithelial carcinoma. Non-HBOC cancer diagnoses: EC endometrial cancer, GC gastric cancer, HNC head and neck cancer.

Genetic testing and variant interpretation were performed at the Department of Molecular Diagnostics, Institute of Oncology Ljubljana (IOL). Next generation sequencing (NGS) was performed on the Illumina MiSeqDx Sequencing System (Illumina, San Diego, CA, USA) with Illumina’s TruSight Cancer Panel or TruSight Hereditary Cancer Panel to enrich and sequence all translated exons and ±25 base pairs flanking intronic regions of 19 HBOC genes (*ATM, BARD1, BRCA1, BRCA2, BRIP1, CDH1, CHEK2, EPCAM, MLH1, MSH2, MSH6, NF1, PALB2, PMS2, PTEN, RAD51C, RAD51D, STK11, TP53*) as described previously [7]. NGS (Fig. 1, patients ID44, ID58, ID63, ID69, ID76, ID77, ID84, ID91, ID97, ID104, ID105) or Sanger sequencing (Fig. 1, patients ID64, ID71, ID73, ID78, ID81-ID83, ID85-ID90, ID92-ID96, ID98-103) were performed on blood samples per clinical geneticists’ discretion and relevant institutional guidelines. Testing for potential large intragenic deletions in *BRCA1* and *BRCA2* genes was performed by utilizing NGS data with copy number analysis using SeqNext v4.4.0 (JSI medical systems) or with multiplex ligation-dependent probe amplification (MLPA) [7]. In carriers, presence of the *BRCA2:c.7892 T* > *C* variant was confirmed using Sanger sequencing from a separate blood sample. In deceased family members with cancer for whom archived formalin-fixed paraffin-embedded (FFPE) material from surgery or biopsy was available, genotyping was preferentially performed on non-tumor tissue; however, when non-tumor tissue was unavailable, tumor tissue was analyzed. FFPE tissue sections were reviewed by a pathologist to determine tumor and non-tumor tissue. Archived FFPE tissue samples were analyzed by Sanger sequencing using a specific primer pair (forward: TTTGTTCAGGGCTCTGTGTG; reverse: TGAAGAAGCACCCTTTCTGG) (Fig. 1; patients ID25, ID42, ID50, ID60, ID66, and ID70) or by NGS using either the AmpliSeq for Illumina BRCA Panel or the TruSight Tumor 170-DNA panel (Illumina) (Fig. 1; patients ID23 and ID48), as previously described [8, 9].

### Framework for VUS reclassification

We first identified the *BRCA2*(NM_000059.4)*:*c.7892T > C germline variant in a young breast cancer patient (Fig. 1, ID91) in 2015, and classified it as a VUS (PM2_Moderate ACMG/AMP (American College of Medical Genetics and Genomics/Association of Molecular Pathology) variant classification criteria criterion was used [10]. After identifying the variant in the proband with metastatic pancreatic cancer, we first performed limited cosegregation analysis with meiosis counting [11] in cancer patients within four known families (Fig. 1). The calculated probability of cosegregation was 1/32 and we were able to reclassify the variant to likely pathogenic in October of 2021 using ACMG/AMP classification criteria PP1_Strong and PM2_Moderate [10, 11]. Only after the initial reclassification to likely pathogenic were unaffected family members offered cascade testing.

In the cosegregation analysis reported herein, the Bayes factor-based quantitative method webtool named COOL, version 3 (Cosegregation Online http://bjfenglab.org/) was used [12]. Through the publicly available online ecclesiastical books of baptisms (https://data.matricula-online.eu/sl/slovenia/), we were able to combine the four initially identified families (Fig. 1) into a single large family (the pseudonymised pedigree is available in Supporting Information 1). Genetically tested and untested family members with and without cancer diagnoses, as well as obligate carriers, were included in the cosegregation analysis; only the least informative family members (young, healthy carriers/non-carriers) [13] were not included. Incidence rates of cancer specific for the population of Slovenia (years 2003-2007) were applied in the COOL analysis. As per our clinical pathway, all anamnestically reported cancer diagnoses are routinely verified against the high-quality Slovenian Cancer Registry, as cancer reporting has been mandatory in Slovenia since 1950. HBOC phenotype was defined as diagnosis of breast, ovarian/tubal/primary peritoneal serous, pancreatic and/or prostate cancers, including non-invasive cancers (DCIS (ductal carcinoma in situ) and STIC (serous tubal intraepithelial carcinoma). For the variant’s reclassification, the *BRCA2* gene-specific ACMG/AMP variant classification criteria, as recommended by the ClinGen (Clinical Genome Resource) Evidence-Based Network for the Interpretation of Germline Mutant Alleles (ENIGMA) consortium’s Variant Curation Expert panel (VCEP), were applied [14].

In accordance with our clinical pathway, participants signed the informed consent for genetic testing and gave permission for their pseudonymised data to be used for research purposes and publishing [15]. We also obtained approval from the Institute’s Committee for Medical Ethics (#ERIDEK-0069/2020).

### Ethics approval and consent to participate

participants have signed the informed consent for genetic testing and have given permission for their pseudonymised data to be used for research purposes and publishing. We obtained approval from the Institute’s Committee for Medical Ethics ERIDEK-0069/2020. The study was performed in accordance with the Declaration of Helsinki.

## Results

### Identification of carriers

In February 2020, a 50-year-old male proband with newly diagnosed metastatic pancreatic cancer was referred to our cancer genetics clinic for fast-track genetic testing for potential treatment with PARP inhibitors. Multigene panel testing on his blood sample identified a rare missense variant *BRCA2*(NM_000059.4)*:*c.7892T > C p.(Leu2631Pro). This variant is located in the DNA-binding domain, and at that time had not been previously reported in the ClinVar database (https://www.ncbi.nlm.nih.gov/clinvar/variation/1005003/) or the Genome Aggregation Database (gnomAD v2.1 and v3.1). Additionally, no other carriers were identified in the Slovenian Genome Variant Browser of 9425 tested individuals [16]. We initially classified the variant as a VUS using the ACMG/AMP criterion PM2_Moderate (absent from general population database GnomAD v2) [10].

However, due to a personal and extensive family history of HBOC-associated cancers (Fig. 1A), we suspected HBOC syndrome. We actively searched for other potential carriers of this variant in the laboratory database of 10028 tested individuals obtained through routine diagnostic practice since 2015. Thus, we identified five additional carriers (Fig. 1, ID58, ID91, ID97, ID104, ID105) with personal history of breast cancer and/or extensive family history of other HBOC associated cancers. According to their anamnestic data, we were able to cluster all six carriers into four distinct pedigrees (Fig. 1), all originating from the same small northeastern geographical area of Slovenia.

No other P/LP variant or VUS in *BRCA2* or other HBOC predisposition genes were identified in any of the tested individuals. Detailed cancer diagnoses, age at test or diagnosis of cancer or death, and *BRCA2*:c.7892T > C carrier status are listed in the Fig. 1.

As the proband was a candidate for treatment with PARP inhibitors, an ad hoc board of experts of clinical geneticists and laboratory bioanalysts from IOL was established. The board reviewed the available data and proposed additional cosegregation analysis in all family members with cancer diagnoses, as well as genetic testing on the available FFPE tumour/non-tumour samples from deceased family members with cancer. The board concluded there is a high probability of the variant being deleterious; however, the reclassification in accordance with the internationally established ACMG/AMP criteria would be a lengthy process. As an exception to standard procedures, the expert board proposed the proband with the VUS in *BRCA2* gene could be offered treatment with PARP inhibitors, if clinically indicated and if consented by the patient.

### Treatment with a PARP inhibitor in proband with metastatic pancreatic cancer

In July 2020, after radiologically confirmed partial response to five cycles of platinum-based chemotherapy, the proband was offered maintenance targeted therapy with a PARP inhibitor, olaparib, and he consented to the treatment. Following four months of therapy with olaparib, complete radiological remission was observed. Throughout, proband reported improvements in wellbeing, appetite, weight gain, and physical activity. After 48 months of maintenance therapy with PARP inhibitors, he was admitted to a local hospital due to epileptic seizure, and two brain metastases were identified. CT scan showed lasting complete response extracranially. Brain metastases were surgically removed, but proband died shortly after due to sepsis.

### Impact of cascade testing on healthy carriers

All identified carriers were offered personalised surveillance and risk-reducing surgeries at the OIL in accordance with local guidelines. At her second check-up, breast cancer was diagnosed in a 41-year-old female carrier (Fig. 1, ID104), and STIC was found after a risk-reducing salpingo-oophorectomy in a 53-year-old carrier (Fig. 1, ID 82).

### Segregation analysis with the COOL v3

Altogether, 71 blood relatives were included in the final cosegregation analysis: 43 genetically tested from blood or FFPE samples as detailed in the “Methods,” 20 obligate carriers, and eight untested individuals (the pseudonymised pedigree is available in the Supporting Information 1). Using the full-likelihood method based web tool COOL v3, the computed overall Bayes factor was 7059923579, and the overall cosegregation LOD score was 9.85 (see Supporting Information 2), suggesting extremely high likelihood of linkage between the HBOC phenotype and *BRCA2*:c.7892C > T variant.

### Reclassification using the ClinGen *BRCA2* VCEP recommendations

Reclassification of *BRCA2*:c.7892T > C using the ClinGen ENIGMA VCEP *BRCA2* gene-specific ACMG/AMP variant classification is detailed in Table 1 [14]. The computed Bayes factor from the extensive cosegregation analysis, updated data from the international database gnomAD, and evidence from published functional studies were included [17, 18]. Evidence codes were applied according to the Criterion Specification Registry (version 1.2.0.; https://cspec.genome.network/cspec/ui/svi/doc/GN097).

**Table 1:** Reclassification of *BRCA2(NM_000059.4)*:c.7892T>C using the ClinGen ENIGMA VCEP *BRCA2* gene-specific ACMG/AMP variant classification from VUS to pathogenic.

Evidence code	Comments
PM2_Supporting	Absent from control population (GnomAD v4)
PS3_Strong	Functional studies show deleterious effect of the variant
PP1_Very Strong	The variant segregates with the disease, with Bayes factor 7059923579 and an overall cosegregation LOD score 9.85

## Discussion

Cosegregation can be a useful and powerful tool for establishing a possible connection between an allele and hereditary cancer predisposition [13, 19], although unspecific phenotype, phenocopies, incomplete age and/or sex-specific penetrance as well as small and scarce families, may present challenges [11–13]. The COOL Bayes factor-based quantitative method shows improved accuracy compared to the meiosis counting method, as it incorporates all the aforementioned cancer specific factors as well as population specific incidence rates (i.e. Slovenia) and allele frequency [12], making it the preferred method for cosegregation analysis according to the ClinGen ENIGMA VCEP [14]. In the cosegregation COOL v3 analysis reported herein, we included 43 genetically tested individuals, 20 obligate carriers and 8 untested individuals—distant relatives with extreme phenotype (i.e. young cancer patients and healthy older family members) [13], and observed a very strong likelihood for the variant’s causality. The publicly available ecclesiastical books of baptisms proved extremely useful and aided us in connecting four initially identified families into a single large family. In this manner, we were able to identify common female and male ancestors born in the specific northeastern region of Slovenia in the first half of the 19th century.

The variant seems very rare and was reported outside this family only three times in the international databases of variants: in a 40–45 year-old male in the UK Biobank [20], in the ClinVar database as VUS by the Labcorp Genetics (formerly Invitae) in March 2021, and as likely pathogenic by the Ambry Genetics in April 2025 (accessed online in 27 October 2025; https://www.ncbi.nlm.nih.gov/clinvar/variation/1005003/).

Two computational meta-predictors, BayesDel (0.2955) and REVEL (0.81), also support the variant’s pathogenicity. Additionally, in 2025, two independent functional studies using CRISPR-Cas9-based saturation genome editing multiplex assay of variant effect (SGE MAVE) both demonstrated deleterious effect of the variant [17, 18]. Moreover, the proband’s favorable response to systemic therapy with platinum agents and a PARP inhibitor could support the possibility that this variant may function as a biomarker for homologous recombination deficiency (HRD). In fact, this therapeutic sensitivity may also be interpreted as in vivo evidence of HRD deficiency.

We observed concordant genotype and phenotype in 26/27 tested family members with HBOC phenotype: only one member with prostate cancer at the age of 54 was not a carrier of the VUS or any other germline VUS or P/LP identified using NGS panel testing. The only non-HBOC-related cancer diagnoses in carriers were a signet-ring cell gastric carcinoma in a female obligate carrier at the age of 60; however, there is emerging data suggesting increased risk of gastric cancer in carriers of P/LP variants in *BRCA2* [21].

Our analysis has several limitations. Cosegregation within a single family can be insufficient proof of causality, as the detected variant may be in physical linkage with another undetected pathogenic variant [11–13]. However, cosegregation within multiple families was not applicable in our case. Although we could not identify any other P/LP variants in *BRCA2* or other known HBOC predisposition genes, some rare types of deleterious variants (structural variants, deep intronic, retrotransposon inclusions) could have been missed due to certain limitations of targeted, short-read NGS testing. Also, not all cancer patients had NGS panel testing performed. Furthermore, tumour tissues from the proband and other patients carrying the variant were not analysed for loss of heterozygosity, a second hit in *BRCA2* gene or HRD. Although we observed an excellent and durable response to olaparib in a single metastatic pancreatic cancer patient, this favourable outcome cannot be extrapolated to other carriers of the variant with cancer diagnoses. Some pancreatic cancer patients with germline P/LP variants in *BRCA2* gene are reported to have a complete radiological response to olaparib, however, further data about the potential specific genotype-phenotype correlation are needed [22].

Cosegregation analysis is time-consuming—in this case, it took a year and a half for initial cosegregation of family members with cancer diagnoses. A similar undertaking was reported to take up to three years by other institutions [2, 23]. In the era of precision medicine, prompt results of germline genetic testing, as well as accurate interpretation of identified variants, are needed for optimal systemic therapy selection in some cancer patients. In metastatic pancreatic cancer patients with germline P/LP variants in *BRCA1* or *BRCA2* genes, treatment with platinum-based chemotherapy regimens and PARP inhibitors offers better clinical outcomes [24]. In 2023, Militello et al. [25] reported no benefit from platinum-based chemotherapy regiment compared to non-platinum ones in 30 pancreatic cancer patients with germline VUS, mostly missense, in *BRCA1* and *BRCA2*. These results further support the notion that the majority of VUS in these genes are non-deleterious variants. Pathogenic missense variants in *BRCA2* gene are rare and are located in the DNA-binding domain or affect splicing [26, 27]. According to the published data, more than 90% of VUS in hereditary cancer-related genes are eventually downgraded to B/LB variants, and only 3—8% are reclassified to P/LP [3, 5]. However, by utilizing a HRD assay test in case of missense VUS in the functional DNA-binding domain, a research group was able to reclassify almost 30% to P/LPV [28]. In two recently published independent functional studies, SGE MAVE had been used to functionally characterize nearly all single nucleotide variants in the *BRCA2* DNA-binding domain [17, 18]. These functional assays generate large-scale data that can directly contribute to variant classification. In the future, comprehensive atlases of all genomic missense variants and their classifications will become available through novel functional tests and/or computational tools, and will enable fast and reliable clinical implementation, complementing clinical observations and cosegregation data.

## Conclusions

Reclassification process of VUS to P/LP or B/LB should follow objective use of internationally recommended classification criteria to ensure universal and appropriate personalised surveillance and risk-reducing surgeries in those with increased risk, while avoiding unwarranted interventions and psychological burden in those without. In the era of mainstreaming and precision medicine, cancer patients with germline VUS in target genes should be referred to clinical geneticists with experience in variant interpretation for further evaluation. If a team of experts in variant interpretation estimates a VUS might be deleterious and initiates further time-consuming analyses for the reclassification of such a VUS, these cancer patients may miss the benefits of targeted systemic therapy. In exceptional cases, they could be offered targeted therapy if clinically indicated and if their informed consent is obtained. Caution should be exercised not to misinterpret and causing harm using inappropriate therapy. A close collaboration between clinical geneticists, laboratory bioanalysts and medical oncologists is needed in such unique cases. Only after completing all necessary analyses and successful reclassification to P/LP should at-risk healthy family members be offered cascade testing to guide personalised surveillance and risk-reducing surgeries as recommended by the international guidelines. Further efforts are needed to improve, expedite and unify interpretation of variants, especially VUS in clinically actionable genes.

## Supplementary information

Supplementary info1

Supplementary info1

## Data Availability

The data that support the findings of this study are available on reasonable request from the corresponding author. The data are not publicly available due to privacy or ethical restrictions.
