# Correction: Cosegregation analysis following an excellent response to olaparib in a pancreatic cancer patient carrier of *BRCA2*:c.7892 T > C variant enables its reclassification from VUS to pathogenic

**DOI:** 10.1038/s44276-026-00217-x

**Published:** 2026-04-02

**Authors:** Ksenija Strojnik, Ana Blatnik, Mateja Krajc, Aleksander Novaković, Marija Ignjatović, Janja Ocvirk, Vida Stegel, Petra Škerl, Gašper Klančar, Srdjan Novaković, Vita Šetrajčič Dragoš

**Affiliations:** 1 Department of Clinical Cancer Genetics, Institute of Oncology Ljubljana, Ljubljana, Slovenia; 2 University of Ljubljana, Ljubljana, Slovenia; 3 Division of Medical Oncology, Institute of Oncology Ljubljana, Ljubljana, Slovenia; 4 Department of Molecular Diagnostics, Institute of Oncology Ljubljana, Ljubljana, Slovenia

Correction to: *BJC Reports* 10.1038/s44276-026-00206-0, published online 16 February 2026

During the production process, Table 1 has been omitted.

**Table 1:** Reclassification of *BRCA2(NM_000059.4)*:c.7892T > C using the ClinGen ENIGMA VCEP *BRCA2* gene-specific ACMG/AMP variant classification from VUS to pathogenic.

Evidence code	Comments
PM2_Supporting	Absent from control population (GnomAD v4)
PS3_Strong	Functional studies show the deleterious effect of the variant
PP1_Very Strong	The variant segregates with the disease, with a Bayes factor of 7059923579 and an overall cosegregation LOD score 9.85

The original article has been corrected.

