# Supplementary figures and images for: Cosegregation analysis following an excellent response to olaparib in a pancreatic cancer patient carrier of BRCA2:c.7892 T > C variant enables its reclassification from VUS to pathogenic
Source: BJC Rep. 2026 Feb 16;4:5. doi: 10.1038/s44276-026-00206-0 (PMC12909916; doi:10.1038/s44276-026-00206-0)

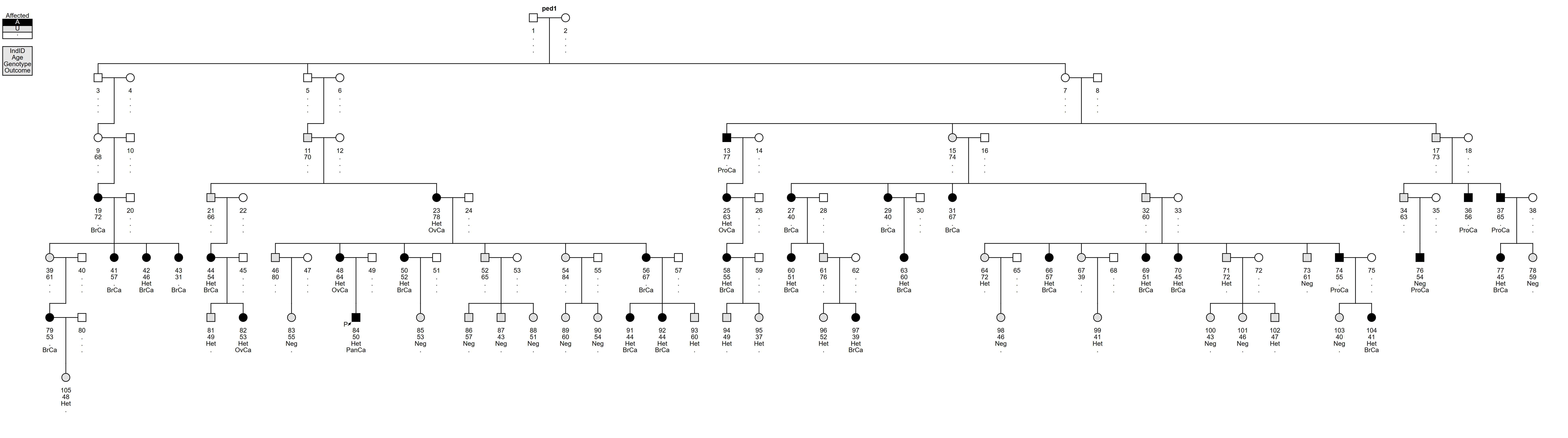

Supplement: Supplementary file 1 — Supplementary info1 [file 44276_2026_206_MOESM1_ESM.png]
