# Supplementary material for: Cosegregation analysis following an excellent response to olaparib in a pancreatic cancer patient carrier of BRCA2:c.7892 T > C variant enables its reclassification from VUS to pathogenic
Source: BJC Rep. 2026 Feb 16;4:5. doi: 10.1038/s44276-026-00206-0 (PMC12909916; doi:10.1038/s44276-026-00206-0)
Supplement: Supplementary file 2 — Supplementary info1 [file 44276_2026_206_MOESM2_ESM.pdf]

Cosegregation OnLine (COOL) v3

Computed by http://BJFengLab.org/

Analysis date: Mon Apr 14 04:12:19 AM MDT 2025
Program version: 2024-03-19
Database version: 2022-11-29
Latest update version: 2022-12-08
URL: http://fenglab-r9.chpc.utah.edu/results/coseg/f5f72658-3266-4099-8b3e-806a7688b301/index.html

Cosegregation Analysis Parameters:

Gene=BRCA2
Pedigree File=Segregation_april2025_FINAL_VERSION.txt
Pedigree IDs=ped1
Variant of interest=
Penetrance File=not_uploaded
RelativeRisk File=not_uploaded
Allele Frequency=0.000001
Mutation Rate=
Population=Slovenia
Year Range=1998-2002
Break Loop=no
Survival Model=yes

Cosegregation Analysis Results:

Overall cosegregation LOD score: 9.8488
Overall Bayes factor: 7059923578.93667198007231817833
